# Supplementary material for: Cardenolide toxin diversity impacts monarch butterfly growth and sequestration
Source: eLife. 2026 Mar 16;14:RP109003. doi: 10.7554/eLife.109003 (PMC12991640; doi:10.7554/eLife.109003)
Supplement: Supplementary file 1. — Chemical shifts with an asterisk correspond to values in CDCl3, whereas non-designated ones correspond to values in CD3OD. Data was compared with published records (El-Askary et al., 1993; Rubiano-Buitrago et al., 2022). [file elife-109003-supp1.docx]

Table S1. 1D NMR assignment of key positions for the identification of 15β-hydroxy-calotropin. Chemical shifts with an asterisk correspond to values in CDCl_3_, whereas non-designated ones correspond to values in CD_3_OD. Data was compared with published records (El-Askary et al. 1993, Rubiano-Buitrago et al. 2022).

| Position | δ_H_ (*J* in Hz) | δ_C_ |
| --- | --- | --- |
| 1 | 1.12 (t,12) | 36.5 |
|  | 2.46 (dd,12,4) |  |
| 2 | 3.92 (m) | 73.1 |
| 3 | 3.88 (m) | 69.8 |
| 15 | 4.51 (dd,9,7)  4.50 (t,7.6)* | 72.9  72.1* |
| 16 | 1.67(m) | 37.6 |
|  | 2.62 (m) |  |
| 17 | 2.7 (dd,10,4.7) | 49.3 |
| 18 | 0.84 (s) | 16.5 |
| 19 | 10.07 (s) | 208 |
| 1' | 4.44 (s) | 97 |
| 2' |  | 91.1 |
| 3' | 3.58 (dd,12,4.9) | 73.7 |
| 4' | 1.54 (m)* | 38.5* |
|  | 1.85 (m)* |  |
| 5' | 3.64 (m)* | 67.7* |
| 6' | 1.23 (d,6.4) | 20.9 |
